# Mechanical Properties of the Periodontal System and of Dental Constructs Deduced from the Free Response of the Tooth

**DOI:** 10.1155/2018/4609264

**Published:** 2018-09-17

**Authors:** Cosmin Sinescu, Virgil-Florin Duma, Dorin Dodenciu, Stefan Stratul, Marius Manole, Gheorghe Eugen Draganescu

**Affiliations:** ^1^Department of Dental Materials and Dental Prostheses Technology, ‘Victor Babes' University of Medicine and Pharmacy of Timisoara, 2 Eftimie Murgu Str., 300041 Timisoara, Romania; ^2^3OM Optomechatronics Group, Faculty of Engineering, ‘Aurel Vlaicu' University of Arad, 77 Revolutiei Ave., Arad 310130, Romania; ^3^Doctoral School, Polytechnic University of Timisoara, 1 Mihai Viteazu Ave., Timisoara 300222, Romania; ^4^Department of Periodontology, ‘Victor Babes' University of Medicine and Pharmacy of Timisoara, 2 Eftimie Murgu Str., 300041 Timisoara, Romania; ^5^Department of Dentistry, ‘Iuliu Hatieganu' University of Medicine and Pharmacy of Cluj-Napoca, 8 Babes Street, 400012 Cluj-Napoca, Romania; ^6^Bioengineering Center, Department of Mechanics and Vibrations, Polytechnic University of Timisoara, 1 Mihai Viteazu Ave., 300222 Timisoara, Romania

## Abstract

The biomechanical behaviour of the periodontal ligament (PDL) is still not well understood although this topic has been studied for almost 100 years. This study reports on clinical and mathematical studies to determine the constitutive law of the PDL. A set of mechanical parameters of the tooth-PDL system is obtained, and a new method for the evaluation of these parameters from the free response of the tooth is introduced. This response is produced by repeated impacts applied to the gingival tissue in the apical part of the tooth investigated—with the aid of a Periotest exciter. A Doppler ultrasound probe is utilized to determine the response of the tooth-PDL system. The parameters evaluated from these measurements can be considered as the elastometric properties of the dental system investigated. A modal analysis/system identification method is utilized to estimate these parameters. The investigations are carried out for different teeth abutments, both with and without a dental bridge/fixed partial prosthesis (FPP). The differences between the responses of the systems in these two cases are determined with the new method proposed. They are discussed with regard to the specific purposes of the FPP. The study demonstrates that this method can provide the dentist with the necessary objective evaluations regarding the properties and health of the tooth-PDL system, as well as of the construct that is obtained after installing a dental bridge.

## 1. Introduction

The periodontal ligament (PDL) system connects the tooth with the alveolar bone [1]. It supports the tooth in contact with compressive masticatory forces. A proper, healthy tooth-PDL tissue system will result in a normal mobility and in a good support of the tooth. However, in time, this system may be affected by periodontal diseases, which induce changes in the tooth mobility, with consequences on the well-being of the patient.

This tooth mobility is an important parameter that has to be evaluated for the current medical practice, as well as for scientific investigations. It is strongly correlated with the mechanical properties of the tooth-PDL tissue system. Its biomechanical behaviour is still not well understood although this topic has been studied for almost 100 years [2–5]. Several devices and methods have been therefore introduced in order to provide quantitative measurements regarding the tooth mobility; they are an alternative to purely empirical tests. One of these devices is the Periotest [6, 7]. This device, which is used in this research as well, applies a series of small mechanical collisions on the tooth and measures the impact pressure on the tooth expressed as an integer score, intuitively defined, with a range between −8 and 50. Another device is the Osstell ISQ [8, 9], based on vibrating the tooth at its resonance frequency. Other devices, for example, based on the piezoelectric actuators, are in development, for adjustable testing regimes on the tooth [10].

These methods are based on the investigation of the function of the frequency response to a harmonic excitation [11]. The resulting vibrations of the tooth are measured using the laser Doppler vibrometry [12, 13]. This method of the function of frequency response is adequate for dental implants, as well as for *in vitro* investigations [14–16]. In all such investigations, a mechanical system with a single degree of freedom (DOF) has been considered; therefore, a single damped oscillator has been used. This approach has the advantage of the simplicity of the mathematical model involved, but it lacks accuracy in modeling precisely the physical phenomenon. Therefore, other investigation methods have also been developed, in which more complex and thus more accurate mechanical models are utilized, with two [17] or four DOFs [18–20]. The latter are taking into account the tooth mass, the head mass, and the mandibular and the maxillary tooth mass.

As it has been considered that these models are still not able to explain the different motions of the tooth in the alveolar bone which are due to the PDL system, continuous mechanical models of the tooth-PDL system have been further developed. These studies have been focused on two main directions:

- Finite element methods (FEMs) are used in order to calculate the different eigenfrequencies of the tooth, considered as an elastic solid [21, 22]. However, these eigenfrequencies cannot be used for practical investigations of the tooth mobility because they are strongly dependent on the links that the tooth has with the gingiva and with the jaw bone.
- Rheological models of the periodontal tissue [23, 24] have developed more precise nonlinear systems [24], as well as isotropic hyperelastic constitutive models [25, 26]. Their main drawback is the difficulty to perform accurate *in vivo* experiments in order to identify the values of the parameters that describe the rheologic models because it is difficult to make precise and reproductive rheological measurements with small devices and in a noninvasive way [27].

Taking into account the aspects above, we have chosen in the present study not a continuous mechanical model, but the method of the impact response of the tooth with multiple DOFs in order to fulfill the aim of this work, i.e., to provide a more accurate, but also a clinically applicable information on the elastic properties of the tooth-PDL tissue. This evaluation is useful for a better understanding of the properties of the periodontal system considered in it entirely, with the remark that the useful number of DOFs that are relevant is a subject of an evaluation as well. The aim of this assessment of the tooth mobility is to offer both researchers and clinicians an indication of the state of health of the PDL system of the patient.

Another important aspect that has to be investigated is the PDL system for the teeth abutments *under dental bridges/fixed partial prostheses (FPPs)*. No evaluations have been performed for such systems until now, to our knowledge, because the abutments and the FPP are difficult to be investigated separately. We thus consider in the first step of this study different individual teeth, and in the second step, the dental bridge/FPP system which unites such teeth. For simplicity, to demonstrate the validity of the investigation method proposed, two teeth separated by a missing one are considered, and then the FPP which unites them.

We have to point out in this respect that by using the Periotest system, only the mobility of a single tooth can be evaluated. There are no records, to our knowledge, about a mobility evaluation method for teeth placed under a bridge, due to the fact that the behaviour of the teeth is different when they are connected. Imagistic methods like radiography or periodontal probing are also useless in this case. The method to be developed is intended to be utilized for such a specific situation, when it is important to monitor (or to evaluate) the mobility of a tooth under a prosthetic construct without taking out that bridge. The scope is to be able to predict the possibility to change the bridge with a new one on the same teeth and also to determine if it is recommended to extract one or some of the teeth involved before taking out the old bridge.

The methodology of this study is to introduce a set of mechanical parameters using the modal analysis method, with a model with several DOFs. A nonperturbative measurement method, based on the impact response of the tooth, is further discussed for the estimation of these parameters. Their usefulness, the efficiency of the related measuring method to assess the mobility of the teeth affected by periodontal diseases, and the effects of the FPP are finally investigated.

In the remaining of this paper, the mechanical models of the PDL system are discussed in Section 2, while the measuring device and method are described in Section 3. The signal processing is pointed out in Section 4.  Section 5 presents the experimental results, while Section 6 discusses them and extracts rules-of-thumb for such a multiparametric analysis.  Section 7 presents the conclusions of the study and directions of future work.

## 2. Mechanical Models of the Tooth-Periodontal Ligament (PDL) Tissue System

The tooth-PDL tissue system represents a continuous deformable solid, with distributed mass, damping, and elasticity. As pointed out above, there are two ways to describe the properties of this deformable solid, each with a corresponding set of parameters which expresses the physical properties of the system investigated: with more precise rheological models or with a simplified description of these properties with discrete mechanical models with *n* DOFs. This latter type of investigation which we utilize in this study is employed in the modal analysis of vibrating systems or in mechanical systems identification [28, 29].

An advantage of such discrete dynamical models is the fact that the free response of the tooth presents one to several damped harmonic components, corresponding to the one or several DOFs of the system. For this reason, we present in this study first the simple model, with a single DOF, but finally focus on the model with multiple DOFs, to model more precisely the physical phenomenon.

**Figure 1:**
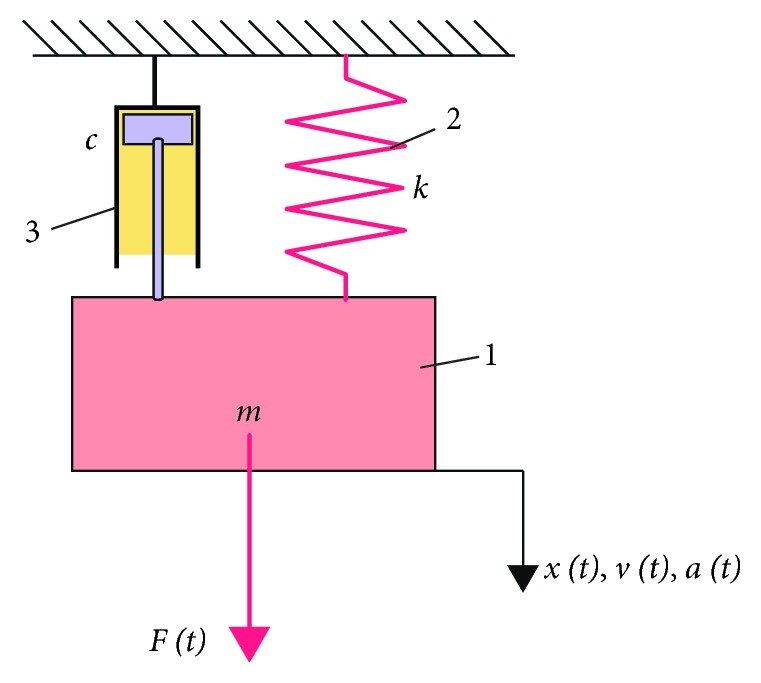
The classical model of a single degree of freedom (DOF) damped oscillator with viscous damping.

- *The classical one-dimensional (1D) vibrational system with viscous damping* consists of a mass *m*, an elastic element with an elastic constant *k*, and a damper characterized by a constant *c* (Figure 1). The system can be subjected to an excitation force *F*(*t*). The classical equation of motion of the mass *m* is

(1) $$\begin{array}{c} m\ddot{x}\left(t\right)+c\dot{x}\left(t\right)+kx\left(t\right)=F\left(t\right), \end{array}$$

where *x*(*t*) is the instantaneous position of the vibrating mass, $v\left(t\right)=\dot{x}\left(t\right)$ is its speed, and $a\left(t\right)=\dot{v}\left(t\right)=\ddot{x}\left(t\right)$ is its acceleration. If an impact is applied to the mass in the absence of the excitation force (i.e., *F* = 0) and for the case of an oscillatory regime (i.e., for *c*^2^ < 4km), the free response of the system can be written in the real or complex form as

(2) $$\begin{array}{c} x\left(t\right)=Ae^{-\xi t}\;\;\sin\left(pt+\phi_{0}\right)=c_{1}e^{\lambda_{1}t}+c_{2}e^{\lambda_{2}t}, \end{array}$$

where *A*, *ϕ*_0_, and *c*_1,2_ are the integration constants.

The parameters of the damped vibrations are the eigenangular frequency *ω*_*n*_, the reduced angular frequency *p*, and the damping factor *ξ*:

(3) $$\begin{array}{c} \omega_{n}=\sqrt{\frac{k}{m}},\\ p=\sqrt{\frac{k}{m}-{\left(\frac{c}{2m}\right)}^{2}},\\ \xi =\frac{c}{2m}, \end{array}$$

as well as the complex pair of eigenvalues

(4) $$\begin{array}{c} \lambda_{1,2}=-\xi \pm \text{i}p, \end{array}$$

where $\text{i}=\sqrt{-1}$ is the imaginary unit.

If one is satisfied with less precise measurements, the free response of the tooth at an impulse excitation may be presented in the form of a damped vibration, Equation (2), which can be characterized by measuring the instantaneous position *x*, the speed *v*, or the acceleration *a* of a point of the tooth. The other two functions can be determined from the one that is measured.

**Figure 2:**
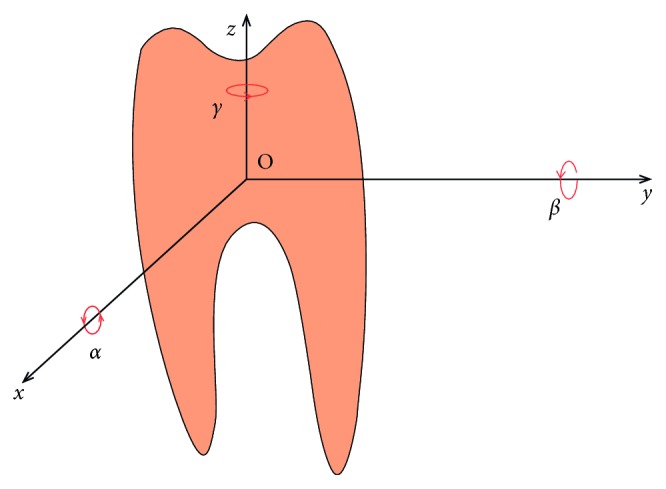
Possible elementary motions of the tooth (considered as a rigid body): the displacements (*x*,*y*,*z*) of its center of mass O and the corresponding rotations (*α*,*β*,*γ*) of the tooth.

One may see that the properties of the tooth-PDL tissue system can be expressed by different pairs of parameters: (*ξ*,*p*), (*ξ*,*ω*_*n*_), or (*c*,*k*). They all can be a measure of the integrity (i.e., of the health state) of the tooth-PDL systems and can thus be added to the score parameter measured with the Periotest device in order to improve the evaluation of such systems.

(ii) To achieve more accurate measurements, the impact response (the “free response”) of the tooth has to contain several components; therefore, *a model with n DOFs* has to be used. Specifically, if we consider that the tooth is a free rigid body, it has six DOFs corresponding to its six possible independent motions: three translations of the center of mass along the axes (*x*, *y*, *z*) and three rotations around these axes, characterized by the angles (*α*, *β*, *γ*) (Figure 2). Actually, because the tooth is an elastic deformable solid, additional motions exist as a result of its flexural and torsional motions; these motions have been investigated by using FEM on simplified models [21, 22].

On the contrary, the tooth is also subjected to constraints that actually reduce its possible motions and its number of DOF. For example, translations on the *x*-axis and *y*-axis, as well as *γ* rotations (i.e., with regard to the *z*-axis) are intuitively not possible. The three remaining movements should be possible—with certain parameters. The discussion on these aspects is completed in Section 6.

This brief analysis shows that one should describe the tooth-PDL system as a linear, coupled vibrating system with *n* DOFs—where a probable useful value, as pointed out above, is *n* which equals 3 and with viscous damping. It is characterized, in a generalization of the discussion on the model (i), by a classical system of second-order differential equations:

(5) $$\begin{array}{c} \left[m\right]\left\{\ddot{q}\right\}+\left[c\right]\left\{\dot{q}\right\}+\left[k\right]\left\{q\right\}=\left\{F\right\}, \end{array}$$

where {*q*}={*q*(*t*)} is the *n*-dimensional column matrix of the generalized coordinates; {*F*}={*F*(*t*)} is the *n*-dimensional column matrix of the excitation forces; [*m*] represents the *n* × *n*-dimensional matrix of inertia (in our case a diagonal matrix, which contains the masses and the moments of inertia); [*c*] is the damping *n* × *n* symmetric matrix; and [*k*] is the stiffness matrix, which is also *n* × *n* symmetric. The symmetry of these matrices results from the action-reaction principle.

System (5), which represents *n*-coupled oscillations is therefore expressed as follows:

(5a) $$\begin{array}{c} \left[\begin{array}{ccccc} m_{1} & 0 & 0 & \ldots & 0\\ 0 & m_{2} & 0 & \ldots & 0\\ 0 & 0 & m_{2} & \ldots & 0\\ \ldots & \ldots & \ldots & \ldots & \ldots \\ 0 & 0 & 0 & \ldots & m_{n} \end{array}\right]\left\{\begin{array}{c} {\ddot{x}}_{1}\left(t\right)\\ {\ddot{x}}_{2}\left(t\right)\\ {\ddot{x}}_{3}\left(t\right)\\ \ldots \\ {\ddot{x}}_{n}\left(t\right) \end{array}\right\}+\left[\begin{array}{ccccc} c_{11} & c_{12} & c_{13} & \ldots & c_{1n}\\ c_{21} & c_{22} & c_{23} & \ldots & c_{2n}\\ c_{31} & c_{32} & c_{33} & \ldots & c_{3n}\\ \ldots & \ldots & \ldots & \ldots & \ldots \\ c_{n1} & c_{n2} & c_{n3} & \ldots & c_{nn} \end{array}\right]\left\{\begin{array}{c} {\dot{x}}_{1}\left(t\right)\\ {\dot{x}}_{2}\left(t\right)\\ {\dot{x}}_{3}\left(t\right)\\ \ldots \\ {\dot{x}}_{n}\left(t\right) \end{array}\right\}+\left[\begin{array}{ccccc} k_{11} & k_{12} & k_{13} & \ldots & k_{1n}\\ k_{21} & k_{22} & k_{23} & \ldots & k_{2n}\\ k_{31} & k_{32} & k_{33} & \ldots & k_{3n}\\ \ldots & \ldots & \ldots & \ldots & \ldots \\ k_{n1} & k_{n2} & k_{n3} & \ldots & k_{nn} \end{array}\right]\left\{\begin{array}{c} x_{1}\left(t\right)\\ x_{2}\left(t\right)\\ x_{3}\left(t\right)\\ \ldots \\ x_{n}\left(t\right) \end{array}\right\}=\left\{\begin{array}{c} F_{1}\left(t\right)\\ F_{2}\left(t\right)\\ F_{3}\left(t\right)\\ \ldots \\ F_{n}\left(t\right) \end{array}\right\}. \end{array}$$

In the absence of the excitation force, Equation (5) becomes

(6) $$\begin{array}{c} \left[m\right]\left\{\ddot{q}\right\}+\left[c\right]\left\{\dot{q}\right\}+\left[k\right]\left\{q\right\}=\left\{0\right\}. \end{array}$$

Its solution gives *the free response* (i.e., *the response to a Dirac impulse*) of the system:

(7) $$\begin{array}{c} \left\{q\right\}=\left\{\begin{array}{c} a_{1}\\ a_{2}\\ \ldots \\ a_{n} \end{array}\right\}{\text{e}}^{\lambda t}, \end{array}$$

where *a*_*μ*_, *μ*=1, *n* are the complex quantities.

Equation (5) gives *n* solutions for the *n* complex conjugate pairs of eigenvalues *λ*_*μ*_ and eigenvectors {*a*_*μ*_}, where *μ*=1,...,*n*. These eigenvalues and eigenvectors are the solutions of the matrix equation:

(8) $$\begin{array}{c} \left(\left[m\right]\lambda^{2}+\left[c\right]\lambda +\left[k\right]\right)\left\{a\right\}=\left\{0\right\}. \end{array}$$

The eigenvalues *λ*_*μ*_ are characterized by *n* complex conjugate pairs because, similar to the model (i),

(9) $$\begin{array}{c} \lambda_{\mu }=-\xi_{\mu }\pm ip_{\mu }. \end{array}$$

It is well-known that the free response can be written as a combination:

(10) $$\begin{array}{c} x\left(t\right)=\overset{n}{\underset{\mu =1}{\sum }}A_{\mu }\;\;\exp\left(-\xi_{\mu }t\right)\cdot \cos\left(p_{\mu }t+\phi_{\mu }\right)=\overset{n}{\underset{\mu =1}{\sum }}\left[c_{\mu }\;\;\exp\left(\left(-\xi_{\mu }+\text{i}p_{\mu }\right)t\right)+d_{\mu }\;\;\exp\left(\left(-\xi_{\mu }-\text{i}p_{\mu }\right)t\right)\right], \end{array}$$

where the pairs (*A*_*μ*_,*ϕ*_*μ*_) or (*c*_*μ*_,*d*_*μ*_) represent the integration constants.

The superposition of the free responses of the *n*-independent damped oscillations (Equation (10)) with motion equations given by Equation (9) and characterized by the same eigenvalues *λ*_*μ*_ finally produces the following:

(11) $$\begin{array}{c} M_{\mu }{\ddot{X}}_{\mu }\left(t\right)+C_{\mu }{\dot{X}}_{\mu }\left(t\right)+K_{\mu }X_{\mu }\left(t\right)=0,\;\mu =1,...,n, \end{array}$$

where *X*_*μ*_ are the modal coordinates and *M*_*μ*_,*C*_*μ*_,and*K*_*μ*_ represent the modal mass, the modal damping coefficient, and the modal elastic constant, respectively.

Finally, the mechanical parameters of the free *n*-dimensional vibrating system can be expressed in three different ways: using the damping factors and the pseudoangular frequencies {*ξ*_*μ*_,*p*_*μ*_}, *μ*=1,...,*n*; using the modal parameters {*M*_*μ*_,*C*_*μ*_,*K*_*μ*_}; or using the damping factors and the natural angular frequencies {*ξ*_*μ*_,*ω*_n*μ*_}, the latter equivalent to {*ξ*_*ν*_,*p*_*ν*_}. These parameters are defined for each of the *n* DOFs, similar with the definitions for a single DOF given by Equation (3).

## 3. The Measuring Device and Method

In order to measure experimentally the elastic parameters {*ξ*_*μ*_,*p*_*μ*_}, {*ξ*_*μ*_,*ω*_n*μ*_}, or {*M*_*μ*_,*C*_*μ*_,*K*_*μ*_}, where *μ*=1,...,*n* of the tooth-periodontal tissue system defined in the previous section, we introduce a method for measuring *the free response* of this periodontal system.

**Figure 3:**
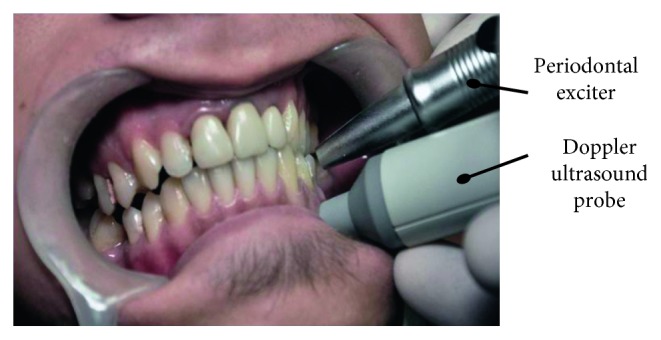
Positioning of the Periotest and of the Doppler ultrasound sensor towards an investigated tooth.

To achieve this, we utilize a repetitive impact exciter represented by a Periotest device in order to periodically apply short impulses normal to the crown region of the teeth, as shown in Figure 3. The PDL is a complex structure which consists of fiber bundles, fluids, cells, and vessels; it displays a time-dependant and force-dependant, nonlinear behaviour. The force/velocity regime of a clinical situation is typically low to medium speed, high force, but in the present study, we chose an experimental protocol which is common for the Periotest, i.e., with a higher speed and a lower force, in order to provide a significant response from the tooth. The mass of the excitation head of the Periotest device is *m* = 8 g and the time of impact is *τ* = 0.1 to 0.2 ms, with a repetition frequency of 4 Hz. All these parameters are also common for such tests. In order to insert the Doppler system in the same position when the measurements are done, we used a plastic individual dental guard extended in the investigation zones (i.e., in the apical regions) with a 5 mm support channel.

**Figure 4:**
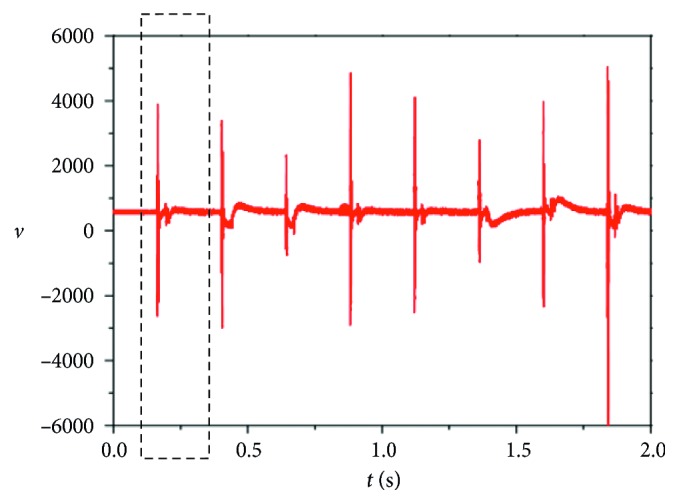
Typical sequence of the measured response to the Periotest pulses (where the instantaneous velocity *v*(*t*) is in arbitrary units).

The free response of the tooth to the impacts was recorded with a Doppler ultrasound sensor-type Mini Dopplex D900 (Huntleigh Healthcare Inc., Cardiff, UK), applied to the apical area of the tooth investigated. This analog device measures the instantaneous speed of the tooth (Figure 4), with an analog voltage as the output signal. An advantage of this measuring method is that the Doppler ultrasound device does not interact mechanically with the tooth-PDL system; the measuring method is thus a contactless one. The analog output signal from the Doppler ultrasound sensor is introduced in a PC with a professional sound card-type Creative AUDIGY 2 which operates on 16 bits with a sampling frequency of 44.1 kHz.

## 4. Signal Processing and Identification Procedure: The Prony Series Method

We consider the system as a linear vibrational one with viscous damping and *n* degrees of freedom. Its impact response measured in a point can be written classically as the linear combination of 2*n* terms of complex argument exponentials:

(12) $$\begin{array}{c} x\left(t\right)=\overset{2n}{\underset{\nu =1}{\sum }}A_{\nu }e^{\lambda_{\nu }t}. \end{array}$$

The modal analysis/structural dynamics provides a series of methods (in time or in the frequency domain) that are able to extract the relevant information concerning the properties of the physical system from the experimental results [28, 29]. In the presence of noise or of a reduced number of measured values, nonparametric methods like the autoregressive or the minimum variance method have to be utilized [27, 30]. In this study, we utilize the Prony series method because of its capability to provide accurate results from the free response. In comparison to other methods, it also has the advantage that it does not require a response signal with numerous precision points. The algorithm of this Prony series method is presented in the following part of this section [31].

The function *x*(*t*) is the impulse response signal of the system, which represents the acceleration, the speed, or the instantaneous position measured in a point of the system. We consider that the recorded signal is sampled with a sampling frequency *ν*_s_ in accord with Shannon's theorem; thus, the sampling frequency obeys the condition *ν*_s_ ≥ 2*ν*_max_, where *ν*_max_ represents the maximum frequency of the signal. The sampling frequency is given by Δ*t*=1/*ν*_s_.

The sampled signal represents the signal at the discrete equidistant moments of time *t*_*k*_=*k*Δ*t*:

(13) $$\begin{array}{c} x_{k}=x\left(t_{k}\right),\;k=0,1,2,...,N-1, \end{array}$$

where *N* represents the number of points in which the signal is recorded.

The recorded response given by Equation (12) can then be expressed in the sampled form:

(14) $$\begin{array}{c} x_{k}=\overset{2n}{\underset{\nu =1}{\sum }}A_{\nu }e^{\lambda_{\nu }k\Delta t}, \end{array}$$

where *λ*_*ν*_ are unknown complex conjugate quantities, depending on the damping factors and on the pseudoangular frequencies defined for each DOF similar to Equation (9).

The first step in order to obtain *λ*_*μ*_ is to built the overdetermined system of equations [32, 33]:

(15) $$\begin{array}{c} \left[\begin{array}{ccccc} x_{0} & x_{1} & x_{2} & \ldots & x_{2n-1}\\ x_{1} & x_{2} & x_{3} & \ldots & x_{2n}\\ x_{2} & x_{3} & x_{4} & \ldots & x_{2n+1}\\ \ldots & \ldots & \ldots & \ldots & \ldots \\ x_{2n-1} & x_{2n} & x_{2n+1} & \ldots & x_{4n} \end{array}\right]\left\{\begin{array}{c} s_{2n}\\ s_{2n-1}\\ s_{2n-2}\\ \ldots \\ s_{1} \end{array}\right\}=-\left\{\begin{array}{c} x_{2n}\\ x_{2n+1}\\ x_{2n+2}\\ \ldots \\ x_{4n+1} \end{array}\right\}, \end{array}$$

which can be written in the formal form [*X*]{*s*}=−{*x*}, where [*X*] is a *m* × 2*n* matrix, {*s*} is a 2*n* × 1 column matrix, and {*x*} is a *m* × 1 matrix (where *m* ≥ 2*n*). This represents, if *m* > 2*n*, an overdetermined system of equations. The solution {*s*} of this system will be found by using the method of the least squares:

(16) $$\begin{array}{c} \left\{s\right\}=-{\left({\left[X\right]}^{\prime }\left[X\right]\right)}^{-1}{\left[X\right]}^{\prime }\left\{x\right\}, \end{array}$$

where (…)′ represents the transpose matrix and (…)^−1^ is the inverse matrix operation.

As a second step, with the aid of the solution {*x*}={*s*_1_,*s*_2_,...,*s*_2*n*_}, a 2*n*-degree algebraic equation can be built:

(17) $$\begin{array}{c} z^{2n}+s_{1}z^{2n-1}+s_{2}z^{2n-1}+\cdots +s_{2n}=0, \end{array}$$

which has its solutions dependent on the modal parameters *λ*_*ν*_:

(18) $$\begin{array}{c} z_{\nu }=\exp\left(\lambda_{\nu }\Delta t\right),\;\text{where }\nu =1,2,...,2n. \end{array}$$

The modal parameters finally are obtained as a set of *n* pairs of complex numbers:

(19) 1Δt  ln  zν=Re  λμ±i Im  λμ.

The modal damping factor *ξ*_*μ*_ and the pseudoangular frequency *p*_*μ*_ will therefore be the absolute values of

(20) ξμ=1Δt  Relnzν,pμ=1Δt  Imlnzν,

where *μ*=1,...,*n*. From Equation (3), it is possible to obtain the rest of the modal parameters.

## 5. Experimental Results

In order to study and validate the method, the investigations are carried out in two clinical situations of interest to the practitians. In each of them, the same tooth (i.e., the first right mandibular molar 46) was absent and three metal-ceramic FPPs were made. We have used Ni–Cr Wiron 99 (BEGO, USA) for this prosthesis for the metallic infrastructure. Its thermal dilatation coefficient is 13.8 × 10^−6^ K^−1^ for the 25–500°C temperature interval. IPS d.Sign (Ivoclar Vivadent AG, Principality of Liechtenstein) ceramic layers were deposed on the metallic infrastructures.

The mobility of the periodontal system was evaluated *before* and *after* the insertion of the metal-ceramic FPPs in the oral cavities for all the clinical cases considered. The Doppler response to the Periotest excitation, measured with the Doppler ultrasound sensor—as described in Section 3—is represented by a series of equidistant strongly damped oscillations separated by Δ*t* = 25 ms (Figure 4). They were recorded on a computer in *wav* format with a 16 bits precision and a sampling frequency of 44.1 kHz (Section 3). The impulse response of a tooth (abutment) was extracted from the *wav* files using the Cool Edit Pro 2.0 audio processing software (Syntrillium Software, Informer Technologies, Inc.). In order to ensure the same level of the signal, the normalised function included in the software was used. The signal was processed using Visual Sound Instrument (Version 3.2) from heliso.tripod and Auto Signal 6.0 trial edition (Seasolve Software). An accurate damped response was selected for each case from the series of impulses.

In the second clinical case, three units of metal-ceramic FPPs were inserted in the oral cavity. The investigations were performed after preparing the abutments and after the FPP insertion.

**Figure 5:**
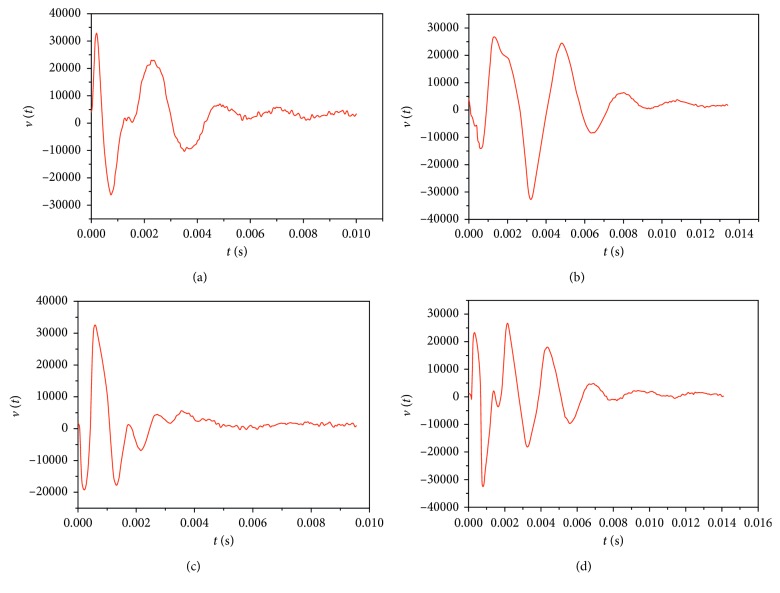
The free response of two blunts for an impact test: blunt 45 (a) without a bridge/FPP and (b) with a bridge/FPP, and blunt 47 (c) without a bridge/FPP and (d) with a bridge/FPP.

In Figure 5, the responses for the abutment 45 and for the abutment 47 are presented, in the case of the blunt: 5(a) without and 5(b) with the bridge. These sequences were utilized to identify the characteristic mechanical parameters. From these sequences, it can be remarked that the signal presents two domains with different forms. The first part (i.e., the 0–2 ms interval) contains the response of the tooth (abutment) during the impact period between the Periotest hammer and the tooth (abutment), followed by the true free response. For the accurate identification of the mechanical parameters from the measured free response, the sequence which contains the interaction between the Periotest impact and the tooth (abutment) had to be eliminated. The remaining part was utilized for the identification procedure with the aid of the Prony series method.

For an accurate application of this method, it is important to know the number *n* of the DOF identified for the system (Equation (15)). This number *n* is determined from the number of peaks that appear in the Fourier spectrum of the signal.

Once *n* is established, the Prony series method is utilized to determine the reduced eigenangulars *p*_*μ*_ and the damping factors *ξ*_*μ*_, where *μ*=1,...,*n*. Each corresponding reduced eigenfrequency is

(21) $$\begin{array}{c} \nu_{\mu }=\frac{p_{\mu }}{2\pi },\;\mu =1,...,n. \end{array}$$

The modal elastic constant and the damping constant are therefore given as follows:

(22) $$\begin{array}{c} k_{\mu }=m_{\mu }\left(p_{\mu }^{2}+\xi_{\mu }^{2}\right),\\ c_{\mu }=2m_{\mu }\xi_{\mu },\;\mu =1,...,n. \end{array}$$

As an option, the density of the elastic constant and of the damping constant can also be defined as the elastic and as the damping constant on the unity of modal mass:

(23) $$\begin{array}{c} \kappa_{\mu }=\frac{k_{\mu }}{m_{\mu }}=p_{\mu }^{2}+\xi_{\mu }^{2},\\ \gamma_{\mu }=\frac{c_{\mu }}{m_{\mu }}=2\xi_{\mu },\;\mu =1,...,n. \end{array}$$

In Tables 1 and 2, the parameters estimated for the abutments 45 and 47, respectively, are presented—for the corresponding records obtained without and with the dental bridge/fixed partial prostheses (FPPs). A clear change of these modal parameters can be remarked; therefore, they can be considered in order to investigate the mobility of the teeth affected by the periodontal diseases [34], as well as the influence of the FPP on this mobility.

## 6. Discussion

The results in Tables 1 and 2 allow for drawing a series of conclusions regarding the tooth-PDL system:

- Three DOFs can be identified in Tables 1 and 2—by using the analysis in Figures 4 and 5—out of the six possible DOFs of the tooth marked in Figure 2. Thus, a confirmation of the initial discussion on the useful number of DOF to be considered—as made with regard to Figure 2—is made. These DOFs refer to both abutments, when considered individually (i.e., without an FPP), and also after the FPP has been installed.
- From the amplitudes *A*_*μ*_ and from the damping factors *ξ*_*μ*_ of these three movements, the active DOFs can be clearly identified:
  - *The β oscillation* (rotation with regard to the *y*-axis, Figure 2) corresponds to the movement with the highest amplitude, as this is the most important movement that the periodontal tissue allows for and as it is in the direction of the impact produced on the tooth (Figure 3). Therefore, in Table 1, for the blunt 45 without an FPP, the *y*-axis oscillatory rotation corresponds to the movement (1), while with an FPP, it corresponds to the movement (2). In Table 2, for the blunt 47 without an FPP, the *y*-axis oscillation corresponds to the movement (2), while with an FPP, it corresponds to the movement (1).
  - *The z-axis translation* (Figure 2) is the movement with the smallest oscillatory amplitudes *A*_*μ*_ and with one of the highest damping factors *ξ*_*μ*_—the latter aspect due to the fact that the rigidity on the *z*-axis is the highest, as this movement is related to the adaptation to the masticatory forces and thus to the necessary stability of each tooth in the alveole of their gum. In both Tables 1 and 2, for the blunts 45 and 47 without and with an FPP, this *z*-axis translation corresponds to the movement (3).
  - *The α oscillation* (rotation with regard to the *x*-axis, Figure 2) corresponds to the movement which has an amplitude that is in-between the minimum and the maximum values above; as this is the movement, the periodontal tissue still allows for a tooth. Therefore, in Table 1, for the blunt 45 without an FPP, the *x*-axis oscillatory rotation corresponds to the movement (2), while with an FPP, it corresponds to the movement (1). In Table 2, for the blunt 47 without an FPP, this oscillation corresponds to the movement (1), while with an FPP, it corresponds to the movement (2).

As it can be seen from the analysis below, from the three movements identified above, it is even enough to use only the first two, i.e., those that are the easiest to point out, as they have the highest and the smallest amplitudes, respectively. The third one may be utilized for a supplemental validation of the conclusions extracted using the previous two.

(iii) By comparing the highest amplitudes *A*_*μ*_ of the two blunts (i.e., those corresponding to the *y*-axis rotation), the blunt 47 has less stronger links to the periodontal tissue, as its oscillations are more significant; therefore, it is a much worst “shape” than the blunt 45 from the point of view of the health of the periodontal tissue.
(iv) After the FPP is in place, the amplitudes *A*_*μ*_ of the *y*-axis rotation grow closer for the two teeth: the one of the blunt 45 increases, while the one of the blunt 47 decreases—as the former stabilizes the latter. The same aspect can be concluded/is confirmed from the *z*-translations, which for the blunt 45 are larger, while for blunt 47 remain approximately constant.
(v) The eigenfrequencies *υ*_*μ*_ are higher for a healthy periodontal tissue, which acts as a strong spring for the system, and are smaller for an affected periodontal tissue, which acts as a weaker spring. However, such a comparison could be done only between the same blunts of a certain person—in time. In the case of the two blunts considered, i.e., 45 and 47, they are different; thus the configuration of their equivalent system (as considered, simplified, in Figure 1) is different. Therefore, one cannot compare their state of health based on the values of their *υ*_*μ*_, but only based on the values of their *A*_*μ*_, as performed above. It can actually be remarked that the eigenfrequencies are higher for the blunt 47 with regard to the blunt 45—due to this different configuration of their systems.
(vi) However, regarding the previous aspect, one can successfully compare the situation of the blunts before and after placing the FPPs: for the blunt 45, the values of *υ*_*μ*_ decrease, while for the blunt 47, they decrease. Again, like for the amplitudes *A*_*μ*_, an average of the initial values is achieved after placing the FPPs, in accordance with the conclusion obtained in point (iv).
(vii) Using the values of the eigenfrequencies *υ*_*μ*_, one can make a comparison between the different DOFs but, again, for the same tooth. Thus, from both tables, the *z*-axis translation has not only the smallest oscillatory amplitudes *A*_*μ*_, but also the highest eigenfrequencies *υ*_*μ*_. This is also a confirmation of the choice of this DOF; as on the *z*-axis, the rigidity of the tooth-tissue system is the highest one, as an adaptation to the maximum forces applied to the system in this direction (i.e., for the masticatory process).
(viii) The modal damping factor *ξ*_*μ*_ is less relevant for the analysis; as by comparing the values in Tables 1 and 2, some of them increase and some decrease after the FPP is placed—with regard to a different DOF. However, by using the composed parameter *K*_*μ*_ defined by Equation (22), the analysis can be completed successfully, as one may see that this parameter follows the trend of the eigenfrequencies *υ*_*μ*_—for each blunt and case; therefore, the same conclusions as at points (v) and (vi) can be reached.
(ix) A confirmation of the entire analysis made above can be made using the third DOF, i.e., *the α oscillation* (rotation with regard to the *x*-axis, Figure 2): *A*_*μ*_ is again higher for the blunt 47 with regard to the blunt 45; after placing the FPP, it increases for 45 and it decreases for 47, thus reaching a sort of average of the initial values. For *υ*_*μ*_ and for *K*_*μ*_, we have already seen that they have the same behaviour as for the other two DOFs. Therefore, in performing such an analysis, one may use only two or even one of the DOF considered, while the other(s) can be used to confirm the results obtained—as it has been seen from the above discussion.

As a remark, one can see that similar conclusions can be reached by analysing the modal damping factor *ξ*_*μ*_ and the damping constant on the unity of modal mass *γ*_*μ*_ (the latter in the last column of Tables 1 and 2), as these two parameters are proportional (Equations (22) and (23)). Therefore, only one of these two parameters is enough to complete the analysis. However, such a simplifying remark cannot be fully made for *K*_*μ*_ (in order to completely eliminate it from the analysis) as this parameter is (Equation (22)) a combination of the parameters *υ*_*μ*_ and *ξ*_*μ*_. Still, as pointed out in (viii), *K*_*μ*_ and *υ*_*μ*_ do have the same trend.

In order to make a clinical validation of the results in this study, we did a mobility evaluation of the teeth 45 and 47 with the Periotest (which is dedicated to such an evaluation) and with the Doppler system—before the application of the bridge. The Periotest is thus giving initial information on which tooth is in a good shape and which is in a bad one. The Doppler evaluation made for the same teeth placed under the bridge gave results of the same type, so the two results of the methods, the classical, clinical (but approximate one) and of the more accurate (but more elaborate) method developed in this study confirm each other.

## 7. Conclusions

We have introduced a linear mechanical model with several DOFs, consisting of a viscous-damped vibrating system. The model was able to describe the mobility of the tooth-PDL tissue system with or without a dental bridge/FPP, as well as to complete the evaluation of each dental bridge abutment.

For the physical modal parameters of the mechanical model, we have used the modal elastic constants and the modal damping constants *k*_*μ*_ and *c*_*μ*_, respectively, where *μ*=1,...,*n* (where *n* is the number of DOFs of the system), or the modal reduced frequency and the modal damping factor *p*_*μ*_ and *ξ*_*μ*_. These can be considered the *elastometric* parameters which characterize the status of the tooth-PDL tissue system [32, 33], affected in a certain degree by parodonthosis [34]. An experimental method based on the free response analysis was utilized in order to obtain these parameters. Using a Doppler ultrasound sensor placed on the gingiva in the vicinity of the tooth investigated, this method eliminated the interaction between the exciter and the tooth-PDL tissue system. The identification of the modal parameters has been performed using the Prony series method because for other methods a response signal with numerous points would have to be used.

As demonstrated in the examples considered, these parameters can be utilized to analyse and to compare the status of the teeth affected by the periodontal disease and what happens after the FPP is set in place. To complete this analysis, an identification of the most significant DOF/movements of the teeth can be easily made first. As demonstrated, the results of the analysis when considering the different DOF/movement possibilities confirm each other—for both teeth considered, both without and with the FPP. In the latter case, the expected average of the parameters is reached, as the healthier tooth losses some of the quality of the tooth-tissue system, while the less healthier tooth gains some of this quality. The stronger tooth-PDL system thus supports the weaker. In conclusion, the results of the model are in good accordance with the physical phenomena. These parameters and their analysis can be thus added to the empirical score parameter measured with the Periotest device, which is useful, but provides too little detail on the situation of the health of the tooth-periodontal system.

Future work in our groups includes the study of the variation of these parameters with the repeated application of the impacts, which constitutes the so-called adaptation phenomenon. An optimization of the testing parameters can also be performed, once the study methodology was determined in the present study. Finally, a distinct direction of work refers to applying imaging methods, including noninvasive ones like optical coherence tomography (OCT) in order to evaluate *in vivo* the health of the periodontal pockets with dedicated handheld scanning probes [35, 36].

## Figures and Tables

**Table 1:** Elastometric parameters of the 45 tooth-PDL tissue system—with and without an FPP.

Blunt 45						
Case	*n*	*ν*_*μ*_(Hz)	*A*_*μ*_	*ξ*_*μ*_(s^−1^)	*κ*_*μ*_	*γ*_*μ*_
Without FPP	1	286.4067	1.097711	337.4217	3352220	674.8434
	2	852.1179	0.2212718	993.2421	2.965201*E* + 07	1986.484
	3	2499.865	0.2102088	1514.978	2.490086*E* + 08	3029.956

With FPP	1	341.1231	1.61918	427.1722	4776381	854.3445
	2	1183.321	1.715744	4200.505	7.292389*E* + 07	8401.011
	3	3256.205	0.8393298	6296.236	4.582272*E* + 08	12592.47

**Table 2:** Elastometric parameters of the 47 tooth-periodontal tissue system—with and without an FPP.

Blunt 47						
Case	*n*	*ν*_*μ*_(Hz)	*A*_*μ*_	*ξ*_*μ*_(s^−1^)	*κ*_*μ*_	*γ*_*μ*_
Without FPP	1	495.2435	1.051082	420.4894	9859532	840.9789
	2	1091.073	3.355402	3601.523	5.996768*E* + 07	7203.047
	3	1559.037	0.3151164	1231.853	9.747362*E* + 07	2463.707

With FPP	1	291.2956	1.092437	371.1869	3487646	742.3738
	2	837.9702	0.8992419	1667.244	3.050121*E* + 07	3334.488
	3	1459.554	0.3104216	1741.271	8.71328*E* + 07	3482.542

## Data Availability

Readers can access the data underlying the findings of the study (please see Figures 4 and 5, as well as Tables 1 and 2).

## References

[1] Beertsen W., McCulloch C. A. G., Sodek J. (1997). The periodontal ligament: a unique, multifunctional connective tissue. *Periodontology 2000*.

[2] Synge J. L. (1933). The theory of an incompressible periodontal membrane. *American Journal of Orthodontics and Dentofacial Orthopedics*.

[3] Fill T. S., Carey J. P., Toogood R. W., Major P. W. (2011). Experimentally determined mechanical properties of, and models for the periodontal ligament: critical review of current literature. *Journal of Dental Biomechanics*.

[4] Keilig L., Drolshagen M., Tran K. L. (2016). In vivo measurements and numerical analysis of the biomechanical characteristics of the human periodontal ligament. *Annals of Anatomy-Anatomischer Anzeiger*.

[5] Nikolaus A., Currey J. D., Lindtner T., Fleck C., Zaslansky P. (2017). Importance of the variable periodontal ligament geometry for whole tooth mechanical function: a validated numerical study. *Journal of the Mechanical Behavior of Biomedical Materials*.

[6] Schülte W., Lukas D. (1992). The Periotest method. *International Dental Journal*.

[7] Aparicio C. (1997). The use of the Periotest value as the initial success criteria of an implant: 8-year report. *The International Journal of Periodontics & Restorative Dentistry*.

[8] Abrahamsson I., Linder E., Lang N. P. (2009). Implant stability in relation to osseointegration: an experimental study in the Labrador dog. *Clinical Oral Implants Research*.

[9] Al-Jetaily S., Al-dosari A. A. (2011). Assessment of Osstell and Periotest systems in measuring dental implant stability (in vitro study). *Saudi Dental Journal*.

[10] Drolshagen M., Keilig L., Hasan I. (2011). Development of a novel intraoral measurement device to determine the biomechanical characteristics of the human periodontal ligament. *Journal of Biomechanics*.

[11] Castellini P., Scalise L., Revel G. M. (2000). Vibration measurements for diagnosis of structural defects on human teeth. *Measurement*.

[12] Castellini P., Scalise L. (1999). Teeth mobility measurement by laser Doppler vibrometer. *Review of Scientific Instruments*.

[13] Göllner M., Holst A., Berthold C., Schmitt J., Wichmann M., Holst S. (2010). Noncontact intraoral measurement of force-related tooth mobility. *Clinical Oral Investigations*.

[14] Cawley P., Pavlakovic B., Alleyne D. N., George R., Back T., Meredith N. (1998). The design of a vibration transducer to monitor the integrity of dental implants. *Proceedings of the Institution of Mechanical Engineers, Part H: Journal of Engineering in Medicine*.

[15] Yamane M., Yamaoka M., Hayashi M., Furutoyo I., Komori N, Ogiso B. (2008). Measuring tooth mobility with a no-contact vibration device. *Journal of Periodontal Research*.

[16] Wijaya S. K., Oka H., Saratani K., Sumikawa T., Kawazoe T. (2004). Development of implant movement checker for determining dental implant stability. *Medical Engineering & Physics*.

[17] Noyes D. H., Solt C. W. (1973). Measurement of mechanical mobility of human incisors with sinusoidal forces. *Journal of Biomechanics*.

[18] Noyes D. H., Solt C. W. (1977). Elastic response of the temporo-mandibular joint to very small forces. *Journal of Periodontics*.

[19] Wang C.-H., Liu H.-W., Ou K.-L., Teng N.-C., Yu J.-J., Huang H.-M. (2008). Natural frequency analysis of tooth stability under various simulated types and degrees of alveolar vertical bone loss. *Proceedings of the Institution of Mechanical Engineers Part H-Journal of Engineering in Medicine*.

[20] Lee S. Y., Huang H. M., Lin C. Y., Shih Y. H. (2000). In vivo and in vitro natural frequency analysis of periodontal conditions: An innovative method. *Journal of Periodontology*.

[21] Xin H., Li Y., Zhao L., Guo W. (2009). Nonlinear finite element analysis of the vibration characteristics of the maxillary central incisor related to periodontal attachment. *Medical and Biological Engineering Computing*.

[22] Chen Y.-C., Tsai H. H. (2011). Use of 3D finite element models to analyze the influence of alveolar bone height on tooth mobility and stress distribution. *Journal of Dental Sciences*.

[23] Gei M., Genna F., Bignoni D. (2002). An interface model for the periodontal ligament. *Transactions of the ASME*.

[24] Natali A. N., Pavan P. G., Carniel E. L., Dorow C. A. (2003). Transversally isotropic elasto-damage constitutive model for the periodontal ligament. *Computer Methods in Biomechanics and Biomedical Engineering*.

[25] Natali A. N., Pavan P. G., Scarpa C. (2004). Numerical analysis of tooth mobility: formulation of a non-linear constitutive law for the periodontal ligament. *Dental Materials*.

[26] Limbert G., Middleton J., Laizans J., Dobelis M., Knets I. (2003). A transversely isotropic hyperelastic constitutive model of the PDL: analytical and computational aspects. *Computer Methods in Biomechanics and Biomedical Engineering*.

[27] Zhurov A. I., Limbert G., Aeschlimann D. P., Middleton J. (2007). A constitutive model for the periodontal ligament as a compressible transversely isotropic visco-hyperelastic tissue. *Computer Methods in Biomechanics and Biomedical Engineering*.

[28] Bereteu L., Draganescu G. E., Stanescu D., Sinescu C. (2011). Quantitative measurement of speech sound distortions with the aid of minimum variance spectral estimation method for dentistry use. *Computer Methods in Biomechanics and Biomedical Engineering*.

[29] Beards C. F. (1996). *Structural Vibration: Analysis and Damping*.

[30] He Z., Fu Z.-F. (2001). *Modal Analysis*.

[31] Greblicki W., Pawlak M. (2008). *Nonparametric System Identification*.

[32] Marple S. L. (1984). *Digital Spectral Analysis with Applications*.

[33] Draganescu G. E., Ercuta A., Bereteu L. (2008). Identification of nonlinear anelastic models. *Journal of Physics: Conference Series*.

[34] Velea O.-A., Sinescu C., Zeicu C., Duma V.-F. (2014). Evaluation of periodontal pockets using different biomaterials: a preliminary study. *Chemistry Magazine*.

[35] Demian D., Duma V.-F., Sinescu C. (2014). Design and testing of prototype handheld scanning probes for optical coherence tomography. *Proceedings of the Institution of Mechanical Engineers, Part H: Journal of Engineering in Medicine*.

[36] Duma V.-F., Dobre G., Demian D. (2015). Handheld scanning probes for optical coherence tomography. *Romanian Reports in Physics*.

